# Effect of titanium versus conventional mini-plate for mandibular angle fractures treatment

**DOI:** 10.6026/973206300210888

**Published:** 2025-04-30

**Authors:** Kuldeep Pal, Swati Tiwari, Sumit Patidar, Rashmi Rai, Anurag Tripathi

**Affiliations:** 1 Department of Oral & Maxillofacial Surgery, Consultant Surgeon, Pal Hospital, Sagar, Madhya Pradesh, India; 2 Department of Oral & Maxillofacial Surgery, Consultants, Dr. Mudgal's Sparsh Hospital and Research, Centre, Gwalior, Madhya Pradesh, India; 3 Department of Oral & Maxillofacial Surgery, Head & Neck Cancer, Reconstructive and Cranio-Maxillofacial Surgeon, Consultant at OraMax Clinic, Indore, Madhya Pradesh, India; 4 Department of Oral & Maxillofacial Surgeon, Consultant Surgical Oncology, Balco Medical Centre, Raipur, Chhattisgarh, India; 5 Department of Oral & Maxillofacial Surgery, Consultant Surgeon, Clinic Oramax Dant Chikitsalaya, Bilaspur, Chhattisgarh, India

**Keywords:** Mandibular angle fracture, titanium miniplate, stainless-steel miniplate, internal fixation, fracture healing, maxillofacial trauma

## Abstract

Both titanium and stainless-steel mini plates were compared for their impact on clinical results after treating 30 patients with fractures at the mandibular angle. People in Group A (with titanium) experienced less pain (VAS), recovered faster from swelling and had a regained higher bite force (320 N) in 3 months compared to Group B (patients in Z-shaped bony plates). Only one case of infection and hardware loosening developed in Group B. Furthermore, bone healing was faster in Group A (in 6.2 weeks) than in Group B (in 7.1 weeks). Thus, titanium mini plates showed the best results.

## Background:

Among maxillofacial trauma injuries, mandibular fractures stand as the most common bone fractures since they affect 36-59% of patients and manifest mainly at the angle of the jaw structure because of their biological and structural design aspects [1, 2]. The primary goal in treating these types of fractures is to achieve restored functionality with minimal secondary effects. The standard surgical treatment for displaced mandibular fractures now includes open reduction and internal fixation (ORIF) with mini plates, as this method allows patients to recover function more quickly with better aesthetics while decreasing the likelihood of malunion [3]. The medical staff chooses conventional stainless-steel mini plates because they provide cost-effectiveness combined with strong mechanical properties. Alternative materials have become necessary because stainless-steel mini plates cause corrosion and allergies and increase the risk of infection [4]. The material capabilities, biocompatibility, corrosion resistance, and low allergenic potential of titanium mini plates have led to their popularity in craniofacial osteosynthesis procedures [5]. The biochemical properties of titanium provide both a high strength-to-weight ratio and osseointegration capability, establishing it as an ideal material for extended surgical implants [6]. The elevated price of titanium implants delivers barriers because they may create economic challenges for communities facing limited resources. Therefore, it is of interest to investigate how titanium mini plates perform compared to regular stainless-steel mini plates when used in the treatment of mandibular angle fractures, examining patient discomfort, the time required for healing, and the occurrence of infections, as well as assessing functional recovery.

## Materials and Methods:

A prospective, randomized clinical study examined 30 patients who received isolated treatment for unilateral mandibular angle fractures.

## Inclusion criteria:

[1] Patients aged 18-50 years.

[2] Isolated unilateral mandibular angle fractures.

[3] Patients requiring treatment through open reduction along with internal fixation of fractures fell under this inclusion category.

[4] Patients reporting within 72 hours of trauma.

## Exclusion criteria:

[1] Comminuted or bilateral fractures.

[2] Operations were excluded from patients who had either diabetes, osteoporosis, or any other systemic condition that negatively influences bone healing.

[3] Patients with a history of allergic reactions to metal implants.

[4] Patients unwilling or unfit for surgery under general anesthesia.

## Patient allocation:

Two separate groups, each comprising 15 patients, were assigned randomly as the selection method for participants. Titanium 2.0 mm mini plates served as the treatment method in Group A patients. The participants in Group B received treatment with 2.0 mm stainless steel mini plates.

## Surgical procedure:

The surgical process took place under general anesthesia through an intraoral method. Ring reduction occurred through manual control before doctors confirmed the results both by sight and through X-ray imaging. A single miniplate extended from the upper border using two screws attached to 2.0 mm four-hole plates placed on either side of the fracture area. The surgical teams performed wound closure through multiple layers, following standardized care instructions for both treatment groups.

## Postoperative evaluation:

The evaluation of patients was conducted at 1 week, 1 month, and 3 months after surgery. The researchers collected the following medical measures during assessments: The evaluation of pain used the Visual Analog Scale (VAS). The clinical assessment of swelling included subjective grading and facial measurements as part of the evaluation. The bite force evaluation required a bite force gauge for measurement. Infection was checked in both subjective and radiographic ways. The examination of hardware stability included both clinical assessment of mobility and radiographic checks for loosening. The process of bone healing were assessed using radiographic images throughout each scheduled follow-up check.

## Statistical analysis:

The statistical analysis was conducted using SPSS version 25.0. The data for continuous variables showed mean values with standard deviations, while categorical variables underwent Chi-square test analysis. The researchers considered a statistical significance when the p-value reached a level of 0.05 or below.

## Results:

A total of 30 patients with unilateral mandibular angle fractures were included in the study, with 15 patients in each group. The mean age of participants was 32.4 ± 8.1 years in Group A (titanium mini plates) and 31.8 ± 7.6 years in Group B (conventional stainless-steel mini plates). There was no statistically significant difference in demographic data between the groups (p> 0.05). At the 1-week follow-up, the mean VAS pain score was significantly lower in Group A (2.1 ± 0.6) compared to Group B (3.6 ± 0.9) (p< 0.05). This difference remained consistent at the 1-month follow-up (Table 1). Postoperative swelling was more pronounced in Group B. At 1 week, facial asymmetry was observed in 9 patients in Group B versus 4 in Group A. Bite force recovery at 3 months was higher in the titanium group (320 ± 40 N) compared to the stainless-steel group (270 ± 35 N), which was statistically significant (p< 0.05) (Table 2). Only one case of postoperative infection was recorded in Group B, which required early hardware removal. No diseases or hardware-related complications were observed in Group A. Bone healing, assessed radiographically, occurred at 6.2 ± 0.5 weeks in Group A and 7.1 ± 0.7 weeks in Group B (p = 0.01) (Table 3).

## Discussion:

Mandibular angle fractures account for a significant segment of facial skeletal injuries, which need surgical management to achieve proper function and attractive results [1, 2]. The medical community has adopted open reduction internal fixation (ORIF) with mini plates as the preferred procedure for treating mandibular angle fractures because it results in both quick functional recovery and secure bone fixation [3, 4]. The therapeutic outcomes of stainless steel and titanium mini plates in mandibular angle fracture treatment, focusing on pain symptoms, swelling and return of general function, infection rates, and hardware stability outcomes. The titanium group presented with reduced postoperative pain, as titanium triggers a more favorable inflammatory response compared to stainless steel, according to previous research [5, 6]. The superior compatibility of titanium contributes to diminished tissue inflammation and reduced discomfort, resulting in lower Visual Analog Scale (VAS) scores in this investigation [7, 8]. Locking miniplates (titanium) allow significantly greater improvements in bite force over-time compared to nonlocking miniplates in mandibular fracture fixation, without compromising other clinical parameters like pain or swelling [9, 10]. Facial swelling is a typical postoperative result; however, its duration of healing becomes shorter when biocompatible materials are employed [11]. 3D miniplates in mandibular fractures are efficacious enough to bear masticatory loads during the osteosynthesis of fractures [12, 13]. The bite force measurement proves to be a reliable tool for evaluating both masticatory efficiency and the progress of bone healing [14]. The titanium implant group demonstrated a more potent biting force at the end of the third month, as they experienced better implant stability and faster functional recovery. Numerous prior studies have shown that titanium implants offer superior biomechanical stability compared to stainless steel, thereby facilitating more efficient rehabilitation [15, 16]. The rates of postoperative infections result from various factors that encompass implant components, surgical approaches, and patient hygiene practices [17]. One infection was reported in the stainless-steel group, while the remaining patients remained infection-free. Studies in the literature confirm that stainless steel tends to develop more corrosion and biofilm formation [18, 19]. Contrary to other materials, titanium creates an oxide layer through which resists both corrosion and bacterial adhesion [20]. The inferior osseointegration of stainless steel leads to hardware loosening more frequently, as indicated by clinical evidence [21]. According to published works, titanium plates demonstrate superior mechanical stability; thus, no hardware loosening occurred within the titanium group [22, 23]. The bones of patients in the titanium group healed at a faster pace compared to patients treated with stainless steel. The combination of titanium and osteo-conductivity led to bone healing at 6.2 weeks in this patient cohort, whereas stainless-steel patients required healing at 7.1 weeks [24, 25].

## Conclusion:

Titanium mini plates generate better medical outcomes by lowering treatment-related issues. The elevated cost of titanium presents a challenge when implementing it in areas with limited financial resources. The field requires additional multicenter studies with larger sample sizes and cost analyses to validate these results and inform medical decisions.

## Figures and Tables

**Table 1:** Comparison of mean VAS pain scores between Groups

**Time Point**	**Group A (Titanium)**	**Group B (Stainless Steel)**	**p-value**
1 Week	2.1 ± 0.6	3.6 ± 0.9	0.003
1 Month	0.8 ± 0.4	1.5 ± 0.6	0.012

**Table 2:** Functional outcomes and swelling post-surgery

**Parameter**	**Group A**	**Group B**	**p-value**
Swelling at 1 Week (Cases)	4	9	0.042
Bite Force at 3 Months (N)	320 ± 40	270 ± 35	0.018

**Table 3:** Postoperative complications and healing time

**Parameter**	**Group A**	**Group B**	**p-value**
Infection (Cases)	0	1	0.301
Hardware Loosening (Cases)	0	1	0.301
Bone Healing Time (weeks)	6.2 ± 0.5	7.1 ± 0.7	0.01
